# SimBA: simulation algorithm to fit extant-population distributions

**DOI:** 10.1186/s12859-015-0525-0

**Published:** 2015-03-14

**Authors:** Laxmi Parida, Niina Haiminen

**Affiliations:** Computational Biology Center, IBM T. J. Watson Research, Yorktown Heights, NY, USA

**Keywords:** Linkage disequilibrium, Population genetics, Subpopulation, Simulation

## Abstract

**Background:**

Simulation of populations with specified characteristics such as allele frequencies, linkage disequilibrium etc., is an integral component of many studies, including in-silico breeding optimization. Since the accuracy and sensitivity of population simulation is critical to the quality of the output of the applications that use them, accurate algorithms are required to provide a strong foundation to the methods in these studies.

**Results:**

In this paper we present SimBA (Simulation using Best-fit Algorithm) a non-generative approach, based on a combination of stochastic techniques and discrete methods. We optimize a hill climbing algorithm and extend the framework to include multiple subpopulation structures. Additionally, we show that SimBA is very sensitive to the input specifications, i.e., very similar but distinct input characteristics result in distinct outputs with high fidelity to the specified distributions. This property of the simulation is not explicitly modeled or studied by previous methods.

**Conclusions:**

We show that SimBA outperforms the existing population simulation methods, both in terms of accuracy as well as time-efficiency. Not only does it construct populations that meet the input specifications more stringently than other published methods, SimBA is also easy to use. It does not require explicit parameter adaptations or calibrations. Also, it can work with input specified as distributions, without an exemplar matrix or population as required by some methods. SimBA is available at http://researcher.ibm.com/project/5669.

## Background

In many studies, it is important to work with an artificial population to evaluate the efficacy of different methods or simply generate a founder population for an *in silico* breeding regimen. The reader is directed to [1] for a review of computer simulations for population and evolutionary genetics. The populations are usually specified by a set of characteristics such as minimum allele frequency (MAF) distribution, linkage disequilibrium (LD) distribution and population fixation indices (*F*_*ST*_ or *G*_*ST*_) (see [2] for detailed descriptions). For instance, in the context of optimizing marker assisted strategies in breeding, the founder population (collection of germplasm lines) could be simulated. Similarly, population of elite lines of small biparental families as in small grain cereals could be simulated for early yield trials, i.e. the stage at which the largest number of individuals with phenotype data are available. Applications of simulation in plant and animal breeding are discussed by [3]. A generative model to simulate the population, i.e., evolving a population over time, is usually rather difficult since the different parameters (such as selection, ancestor population sizes, mutation and recombination rates etc), as well as the “breeding” regimens, are not well understood and almost impossible to estimate effectively. Examples of such methods include those by [4,5]. The non-generative models, on the other hand, do not evolve the population, and the methods often start with a sample population having the desired characteristics and perturb it, either by a regimen of recombinations between the samples or local perturbations. Examples of such methods include [6-8].

EASYPOP [4] and simuPOP [5] are examples of simulators based on an underlying generative model. EASYPOP provides a variety of mating systems and migration and mutation models, while simuPOP can be used to simulate arbitrary non-random mating models. In contrast to these, HapSim [6], SIMLD [7], and epiSIM [8] software are based on non-generative models and simulate the given LD distributions through perturbations. HapSim perturbs an existing haplotype matrix, provided as input, as follows. It models a haplotype as a multivariate random variable with known marginal distributions and pairwise correlation coefficients. First it computes the covariance matrix from an existing sample, and then draws simulated haplotypes from a multivariate normal distribution with that covariance matrix. SIMLD starts with an initial population with the highest possible LD, decaying it over generations through recombinations to fit a desired profile. EpiSIM introduces a notion of Average of Adjacent LD Levels for generating LD patterns, and employs a Markov Chain process to simulate a chromosome. The reader is directed to [9] for a fairly comprehensive list of genetic simulator software systems. The problem we address in this paper is precisely defined as:

### Problem 1.

The task is to generate a stratified population (i.e., with *d* subpopulations) of *n* diploids (or 2*n* haploids) with *m* SNPs that satisfy the following specified characteristics: MAF distribution *p*, LD distribution *r* ^2^ and *F*_*ST*_ or *G*_*ST*_ (stratification) values.

Here we discuss SimBA which is a non-generative approach that has both stochastic and combinatorial components. We model the task as an optimization problem. Based on an algorithm presented in an earlier work [10], we adapt the solution to include multiple simultaneous steps, and introduce the population stratification component. The use of discrete methods enables SimBA to be optimized even in run-time, i.e., the algorithm is linear in the size of the output, thus extremely time-efficient. Additionally, the use of discrete problem-modeling lends unprecedented sensitivity to the algorithms. Subtle changes in the input distributions are observed as corresponding, accurate changes in the output distributions of allele frequency, linkage, and stratification values. Such sensitivity is hard to obtain in perturbation-based non-generative modeling as well as in generative modeling, since the biological processes that give rise to the resulting distributions are generally not well understood. We demonstrate this sensitivity by comparing with results from similar systems from literature. Also, SimBA does not require extensive population-specific parameter tuning or exemplar populations as starting points, unlike most non-generative methods.

## Methods

### Background

To keep the paper self-contained, we recall some basic definitions. Let *p*_1_ and *p*_2_ be the MAF at locus 1 and locus 2 and let *r* ^2^ be the LD between the two loci. Then *D* is defined as follows [11,12]:

(1) $$\begin{array}{@{}rcl@{}} D = \pm r \sqrt {p_{1}(1-p_{1})p_{2}(1-p_{2})}.   \end{array} $$

With a slight abuse of notation we call *D* the LD of the two loci, with the obvious interpretation. Equivalently, the *LD table* of the pairwise patterns, 00, 01, 10, 11, of the two loci, is written as:
(2)


At marker *j*, let *H*_*T*_ be the probability that two random marker values within a substructure are different (without the need for the two to be on the homologous chromosomes of an individual). I.e., the expected heterozygosity at locus *j* for the whole population (T), with MAF $\overline p$, is $H_{T} = 2 \overline p(1- \overline p)$. Wright’s Fixation Index, *F*_*ST*_, for subpopulation *S* is defined as $F_{\textit {ST}} = \frac {H_{T} - H_{S}}{H_{T}} = 1 - \frac {H_{S}}{H_{T}}$. For each subpopulation *s*_*k*_, *k*=1,2,,.., *d*,

(3) $$\begin{array}{@{}rcl@{}} H_{s_{k}} &= &H_{T}(1 - F_{s_{k}T}) \\ &= &\bar{p}(1-\bar{p})(1 - F_{s_{k}T}) \\ &= &2p_{s_{k}}(1 - p_{s_{k}}).  \end{array} $$

Thus $0 \leq p_{s_{k}} = \frac {1 \pm \sqrt {1 - 2H_{s_{k}}}}{2} \leq 1$. Note that $0 \leq H_{s_{k}} \leq 1/2$.

### Constructing stratified populations with given MAF *p*, LD *r*^2^ & *F*_*ST*_’s

**Figure 1:**
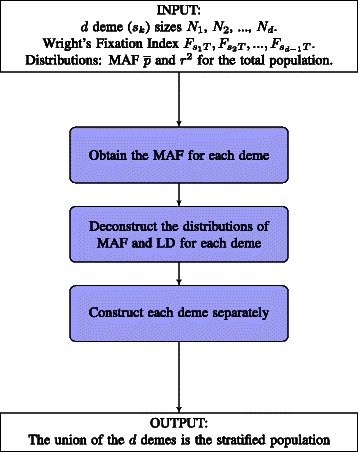
**SimBA flowchart.** Diagram of the overall SimBA simulation approach for stratified populations.

Our approach to building the stratified populations with the given constraints, is to decompose the problem into subproblems, where each subproblem constructs a deme with the desired MAF and LD distribution. The term *deme* and subpopulation are here used interchangeably. An overview of our method is presented in Figure 1.

INPUT:

1. *n*, the total population size and *d* demes with the subpopulation (*s*_*k*_) sizes *N*_1_, *N*_2_, …, *N*_*d*_.
2. MAF $\overline p$ and LD *r* ^2^ distributions for the total population.
3. Wright’s Fixation Index *F*_*ST*_ for *d*−1 demes as $F_{s_{1}T},F_{s_{2}T}, \ldots,F_{s_{d-1}T}$. $F_{s_{d}T}$ is dependent on the specified *d*−1 values, hence not specified as input.

OUTPUT: Matrix *M* where each row is a haplotype and each column is a (bi-allelic) marker.

ALGORITHM OUTLINE:

1. Construct the *d* demes separately as follows.
  - For deme *k*=1,2,..,(*d*−1), compute $p_{s_{k}}$:
    (4) $$\begin{array}{@{}rcl@{}}  p_{s_{k}} &= &\min \left(\frac{1 - \sqrt{1 - 2H_{s_{k}}}}{2}, \frac{1 + \sqrt{1 - 2H_{s_{k}}}}{2} \right) \\ &= &\frac{1 - \sqrt{1 - 2H_{s_{k}}}}{2},  \end{array} $$
    where $H_{s_{k}}$ is $\bar {p}(1-\bar {p})(1 - F_{s_{k}T})$. Then
    $$p_{s_{d}} = \frac {n\bar{p} - \sum_{k=1}^{d-1}N_{k}p_{s_{k}}} {N_{d}}. $$
  - DEME($p_{s_{k}},r^{2}$): Construct each deme, *k*=1,2,..,*d*, with $p_{s_{k}}$ and LD *r* ^2^.
2. Take the union of the *d* demes.

#### Constructing Deme(*p*,*r*^2^)

By convention, the MAF of marker *j*, *p*_*j*_, is the proportion of 1’s in column *j* of *M*. We first deconstruct the distributions to obtain MAF *p*_*j*_ for each marker *j* and $r^{2}_{j_{1}j_{2}}$ for each pair of markers *j*_1_ and *j*_2_. Our approach to constructing the deme is to work with the markers

1. one at a time and
2. without any backtracking.

Note that LD is defined as a pairwise constraint, thus a marker has *m*−1 LD characteristics (constraints) with respect to the other *m*−1 markers. Thus with *m* markers there are a total of *m*(*m*−1)/2 pairwise LD constraints. However, to keep the problem tractable, in practice only up to *k*<<*m* constraints are considered for each marker. The LD constraints are captured as follows. The columns of matrix *M* are constructed in some order *j*_1_,*j*_2_,..,*j*_*m*_. Thus while working on *j*_*s*_, columns *j*_*l*_, *l*<*s* are not altered, but provide constraints. It is critical to traverse the sparse constraint space in a strategic way. Let *l* be the distance between a pair of markers *a*<*b*, i.e., *l*=*b*−*a*. In our implementation, the *l*-distance is picked based on the *r* ^2^ between the pair *a* and *b*, relative to the *r* ^2^ values for the other distances from *b*.

Thus the problem of fitting whittles down to following subproblem, which is used iteratively to span the constraint space and construct the population.

##### Problem 2.

**(** ***k*** **-Constrained Marker Problem (** ***k*** **-CMP))** Given markers (columns) *j*_1_, *j*_2_,., *j*_*k*_, and *r*_1_, *r*_2_, …, *r*_*k*_, and *p*_*k*+1_, the task is to generate column *j*_*k*+1_ with MAF *p*_*k*+1_ such that the pairwise LD with column *j*_*l*_ is *r*_*l*_ (as in Equation 1), *l*=1,2,..,*k*.

### Outline of our approach to solving Problem *k*-CMP

The 1’s in column *j*_*k*+1_ are assigned at random respecting MAF *p*_*k*+1_. Let *D*_*l*_(*j*_*l*_,*j*_*k*+1_) denote the LD between markers *j*_*l*_ and *j*_*k*+1_. Then let the expected value, in the output matrix *M*, be $\overline {D}_{l}(\cdot,\cdot)$. When both the columns fulfill the MAF constraints of *p*_*l*_ and *p*_*k*+1_ respectively, let the observed value be denoted as ${D}^{\text {obs}}_{l}(\cdot,\cdot)$. In other words, if *Q*_10_ is the number of times pattern 10 is seen in these two markers in *M* with *n* rows,

(5) $$ D_{l}^{\text{obs}} = \frac{1}{n} \left({np}_{l}(1-p_{k+1}) - Q_{\tt{10}} \right).   $$

Next, we move the 1’s in column *j*_*k*+1_, such that it simultaneously satisfies *k* conditions, to get a best-fit of ${D}^{\text {obs}}_{l}(j_{l},j_{k+1})$ to $\overline {D}(j_{l},j_{k+1})$. To achieve this, we compare column *j*_*k*+1_ with columns *j*_*l*_, *l*=1,2,..,*k*, that have already been assigned. Thus, first, for each pair of markers *j*_*l*_,*j*_*k*+1_, compute the target deviation, $D_{l}^{\text {target}}$, based on input. Then, move the 1’s in column *j*_*k*+1_ of the output matrix, to get a best-fit to the targets $D_{l}^{\text {target}}$, $D_{2}^{\text {target}}, \ldots, D_{k}^{\text {target}}$ simultaneously.

### Problem *k*-CMP: Hill Climbing

An algebraic approach to obtain an exact solution to *k*-CMP has been discussed earlier [10]. Thus an appropriate value of *k* for a specific class of population(s), can be estimated by using the algebraic method. Here we optimize the hill climbing algorithm presented in [10] by, taking several best simultaneous steps, instead of a single step.

For *l*=1,2,…,*k*, let $G_{l} = {nD}_{l}^{\text {target}}$. Given the input matrix, number of constraints *k*, column *j*_*k*+1_ with MAF $p_{j_{k+1}}$ and target deviations *G*_1_, *G*_2_,…,*G*_*k*_, the hill climbing algorithm is carried out in three steps. For a given *k*, a cost graph ${\mathcal {G}}_{k}$ is built as a pre-processing step. Then, for a given input, the cost graph is instantiated with the input and target signs to obtain ${\mathcal {G}}^{0}_{k}$. Then in Step 2, *s* moves are made to obtain ${\mathcal {G}}^{s}_{k}$. Finally the output (column *j*_*k*+1_) is simply read off ${\mathcal {G}}^{s}_{k}$.

#### Pre-processing: Construct the cost graph ${\mathcal {G}}_{k}$

This step is independent of the input data. For a given *k*, the cost graph ${\mathcal {G}}_{k}$ is defined as follows.

**Figure 2:**
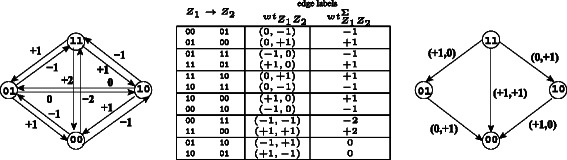
**Cost table and graphs for** ***k*** **=2.** ${\mathcal {G}}_{2}$: The cost graph for *k*=2. To avoid clutter, the *k*-tuple edge labels are not shown on ${\mathcal {G}}_{2}$, but on the edge table in the center. Signed ${\mathcal {G}}_{2}$ with target (+,+) is shown on the right. Note that ${\mathcal {G}}_{2}$ is independent of any data.
  (7) $$\begin{array}{*{20}l} {wt}_{Z_{1}Z_{2}}^{\Sigma} &= &\sum_{l=1}^{k} {wt}_{Z_{1}Z_{2}}[l].  \end{array} $$

Nodes. The cost graph has 2^*k*^ vertices, corresponding to the distinct binary patterns. For example, when *k*=2, the four distinct patterns (and vertices) *Z* are 00, 01, 10, 11.
Edges. A directed edge is introduced between every pair of vertices. We first give the rationale for the cost function, which tracks the LD deviations exactly, for each pair of columns *j*_*l*_ and *j*_*k*+1_, *l*=1,2,..,*k*. The choice of the cost function is critical in evaluating the “climb” in hill climbing process. There are four scenarios as below and the rationale for the cost is discussed in [10]:
  $$\begin{array}{@{}rcl@{}}  c(0,1) &= &-1 \hspace*{15pt} \text{(Scenario I)}\\ c(1,0) &= &+1 \hspace*{15pt} \text{(Scenario II)}\\ c(0,0) = c(1,1) &= &0. \hspace*{15pt} \text{(Scenarios III \& IV)} \end{array} $$
  Using the cost function as defined, the directed edge *Z*_1_→*Z*_2_ is labeled with the *k*-tuple as follows. Figure 2 gives an example for *k*=2.
  (6) $$\begin{array}{*{20}l} {wt}_{Z_{1}Z_{2}} &=&\left(c(Z_{1}[1],Z_{2}[1]), \ldots,\right.\\ &&\left.c(Z_{1}[k],Z_{2}[k])\right) \text{and} \end{array} $$

#### Step 1: Instantiate ${\mathcal {G}}_{k}$ with input data and target to get signed cost graph ${\mathcal {G}}_{k}^{0}$

**Figure 3:**
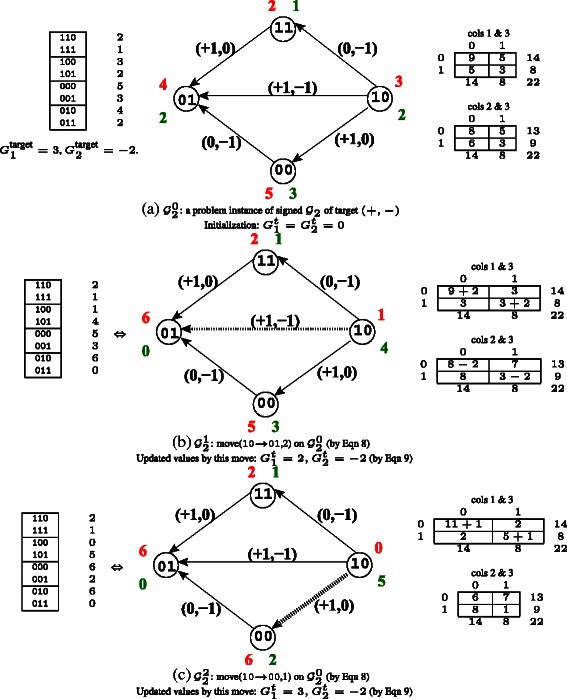
**Hill climbing algorithm example.** The input is shown on the left table in **(a)**. At each step, the two pairwise LD tables are shown on the right. The target of *G*_1_=3 and *G*_2_=−2 is *exactly* met in two moves **(b)-(c)** as shown. The matrix encoded by ${\mathcal {G}}^{2}_{2}$, in (c), is the output.

Given the data, we instantiate the cost graph ${\mathcal {G}}_{k}$, so that it can be traversed. Note that the number of simultaneous constraints being satisfied is *k*. Since the hill climbing regimen never uses an edge in the cost graph that has a negative or zero cost, the signs of the target values determine which edges remain on ${\mathcal {G}}_{k}^{0}$. The cost graph with only edges that have a strict improvement is called the *signed cost graph*. See Figure 3(a) for an example instantiation.

##### Lemma 1.

For any *k*, a signed cost graph is acyclic.

##### *Proof*.

For each of the *k* positions in the *k*-binary pattern, in the signed cost graph, the following transitions are associated with the costs.

$$\begin{array}{@{}rcl@{}} \text{Zero or \(-\)ve cost}: & 0 \overset{0}{\longrightarrow} 0 \overset{-1}{\longrightarrow} 1 \overset{0}{\longrightarrow} 1,\\ \text{Zero or \,+\,ve cost}: & 1 \overset{0}{\longrightarrow} 1 \overset{+1}{\longrightarrow} 0 \overset{0}{\longrightarrow} 0. \end{array} $$

Clearly, if *k*=1, then the signed graph has no cycles. For *k*>1, since the nodes in the signed graph must have distinct *k*-patterns, once a transition is made from 0 to 1, for −ve sign (1 to 0 for + sign respectively), the directed path can not return back to 0 (1 respectively). Hence, there can be no cycles.

#### Step 2: Move at step *s*, to get to ${\mathcal {G}}_{k}^{s}$ from ${\mathcal {G}}_{k}^{s-1}$

Let *A*+=*x* denote incrementing *A* by *x* and *A* −=*x* denote decrementing *A* by *x*. Then Move (*Z*_1_→*Z*_2_,*x*) is defined as follows. For edge *Z*_1_→*Z*_2_, 0≤*x*≤ min(*f*(*Z*_1_),*t*(*Z*_2_)). Move (*Z*_1_→*Z*_2_,*x*) transforms ${\mathcal {G}}^{s}_{k}$ to ${\mathcal {G}}^{s+1}_{k}$ as:

(8) $$\begin{array}{*{20}l} f(Z_{1}), t(Z_{2}) \text{\(-\)= }x \text{ and } f(Z_{2}),t(Z_{1}) \text{\,+\,=} x.  \end{array} $$

(9) $$\begin{array}{*{20}l} {G^{t}_{l}} \text{\,+\,=} \left(x \times {wt}_{Z_{1}Z_{2}}[l]\right), l=1,2,..,k.  \end{array} $$

See Figure 3(b)-(c) for an example of moves. *f*(*Z*) is the number of *Z*0 patterns in the data (shown in red) and *t*(*Z*) is the number of *Z*1 patterns in the data (shown in green).

##### Lemma 2.

At each step *s*, ${\mathcal {G}}_{k}^{s}$ encodes $p_{j_{k+1}}$.

##### *Proof*.

The following can be verified.

1. Let *n* be the total number of samples, *n*_*i*_ be the number of samples with *i* at column *j*, *i*=0,1. Then the following holds for each ${\mathcal {G}}_{k}^{s}$: $ f(Z), t(Z) \geq 0, \sum _{Z} (f(Z) + t(Z)) = n, \sum _{Z} f(Z) = n_{0}, \sum _{Z} t(Z) = n_{1}. $
2. For each node *Z* in ${\mathcal {G}}_{k}^{s}$, the sum *f*(*Z*)+*t*(*Z*) is invariant across all *s*.

Thus it follows that ${\mathcal {G}}_{k}^{s}$ encodes $p_{j_{k+1}}$ at each step *s*.

##### Corollary 1.

$p_{j_{k+1}}$, the MAF at marker *j*_*k*+1_, matches the input exactly.

In the toy example in Figure 3, *n*=22, *n*_0_=14 and *n*_1_=8.

#### Time complexity

The algorithm has two characteristics that make it efficient in time. Firstly, the algorithm does not backtrack, i.e., change the previous *j*_1_, *j*_2_,.., *j*_*k*_ columns, while considering column *j*_*k*+1_. Also note that $|D_{l}^{\text {target}}| \leq p_{j_{k+1}}n$, because the possible range of the deviation from target, $|D_{l}^{\text {target}}|$, for the pair of columns *j*_*l*_,*j*_*k*+1_ is $[0,min(p_{j_{l}}n,p_{j_{k+1}}n)]$. Therefore the number of 1’s that are moved (at most once) is bounded by $p_{j_{k+1}}n$. Secondly, at each column it uses a greedy heuristic of only making a move that results in an improvement in the overall cost. In practice, we observe that these work very effectively in terms of achieving the target accuracies of the solution. The pre-processing step is independent of any data, hence it is computed off-line. Further, the nodes are sorted, using a hash table, by the maximum positive weight of the outgoing edges incident on this node.

##### Instantiating (Time ${\mathcal O}(n)$).

Column *j*_*k*+1_ is scanned once to instantiate cost graph ${\mathcal {G}}_{k}$ with the given *k* conditions to obtain ${\mathcal {G}}^{0}_{k}$. A node *Z* in ${\mathcal {G}}^{s}_{k}$ is defined to be *active* if both *t*(*Z*)>0 and *f*(*Z*)>0. Then:

###### Observation 1.

- The number of active nodes in the initial graph is linear: ${\mathcal {G}}^{0}_{k} \leq n$.
- If node *Z* is not active in the initial graph ${\mathcal {G}}^{0}_{k}$, then *Z* is not active in any of the subsequent graphs ${\mathcal {G}}^{s}_{k}$, for all *s*>0. Similarly, if node *Z* is active in ${\mathcal {G}}^{0}_{k}$, then *Z* is active in ${\mathcal {G}}^{s}_{k}$, for all *s*>0.

In the observation, (a) holds since a node is only active if at least one individual is assigned to it, and there are *n* individuals. Additionally, (b) holds since moves are only made between active nodes, corresponding to the existing individuals’ 0/1 assignments at columns *j*_1_..*j*_*k*_; a node can not become active since we are not changing the values at columns *j*_1_…*j*_*k*_ in the process of generating column *j*_*k*+1_. By the above observation, it takes linear time to instantiate the problem. Additionally, a hash table is used to store the elements that can be accessed in constant time.

###### Lemma 3.

Each problem instance, handling one marker, is optimized in time ${\mathcal O}(n)$.

###### *Proof*.

Since the signed cost graph has no cycles, by Lemma 1, no move is ever undone in the hill climbing regimen. Each cell in column *j*_*k*+1_ is touched no more than twice (once as *from* and once as a *to*). At each move, the candidate cell is obtained in ${\mathcal {O}}(1)$ time, since a hash table is used to store the elements. Thus each marker is handled in time ${\mathcal {O}}(n)$.

## Results and discussion

We implemented the single-step hill climbing method described in [10], at each step choosing the move from the space of all possible moves that most improves the target fit. Although an individual can potentially be touched several times in the process of generating one column, in our experiments we have found each column to be generated in fewer than n moves. We also implemented the subpopulation construction described in Methods. Our hill climbing and subpopulation algorithms are collectively called SimBA.

### Stratified population construction

We evaluated the methods on real human MAF and LD data provided by the International HapMap Project [13], in the LD Data collection: African ancestry in Southwest USA “ASW”, Han Chinese in Beijing, China “CHB”, Luhya in Webuye, Kenya “LWK”, Japanese in Tokyo, Japan “JPT” populations. For one set of experiments we used the *phasing data*, evaluating the ability of SimBA to reconstruct a population when a population with those constraints is known to exist. For another set of experiments, we used averaged *linkage disequilibrium data*, evaluating the ability of SimBA to deconstruct distributions into constraints, and then reconstruct a population with those constraints.

**Figure 4:**
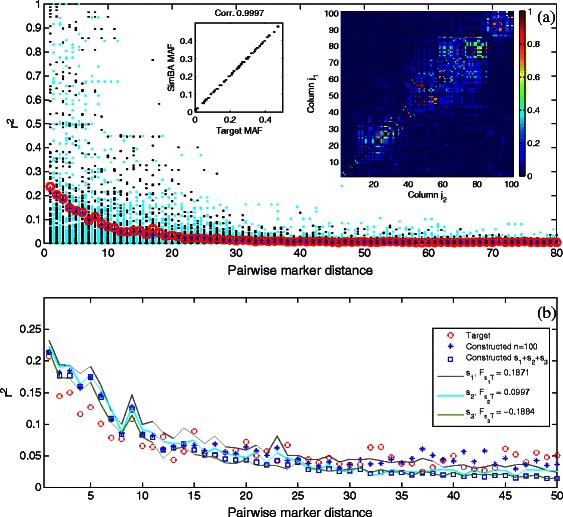
**SimBA simulation results.** **(a)** SimBA hill climbing algorithm for JPT/CHB population with *k*=10. LD fit, MAF fit, and heatmap of LD for each pair of columns (upper left triangle is the target and lower right triangle is the constructed). The LD fit shows the target “o” and constructed “*” mean *r* ^2^ per distance, while the black dots show target and cyan dots constructed *r* ^2^ distribution per distance. **(b)** Constructing three subpopulations with *F*_*st*_ constraints $F_{s_{1}T}=0.2$ and $F_{s_{2}T}=0.1$. MAF and *r* ^2^ constraints from ASW population. Population size *n*=400 constructed as subpopulations with sizes *n*_1_=200,*n*_2_=100,*n*_3_=100. Here *k*=7, distances 1–6 and *j*−1 are used per column *j*. Stars denote constructing a single population without *F*_*st*_ constraints, squares denote the combined population with three subpopulations constructed with *F*_*st*_ constraints. Each subpopulation is also shown separately.

SimBA works by first deconstructing the given LD and MAF distributions into individual values and then does a best-fit of these values. In order to separate the confounding influence of the two, we first evaluated the best-fit component of the method. For this, we generated the test cases as follows. For the experiment shown in Figure 4(a) we applied our algorithm using the HapMap JPT/CHB *phasing data* as input, selecting the first *m*=100 biallelic markers from chr 22, and then using their allele frequencies *p* as the MAF targets, and their pairwise *r* ^2^ values as the LD targets. Using the nearest *k*=10 distances gave excellent results closely fitting the target distributions, as demonstrated by the figure. Figure 4(a) also shows the exact target and constructed *r* ^2^ distributions per distance. In these figures, the target *r* ^2^ for each column and each distance is met with great fidelity, thus demonstrating not only fitting the average *r* ^2^ per distance, but the exact targets, as shown by the heatmap.

In the experiment shown in Figure 4(b), we evaluated the ability of the algorithm to deconstruct the distributions and then carry out the best-fit (i.e., it is not clear that an exact solution matrix exists, due to numerous possibly conflicting *r* ^2^ constraints as input). We binned the ASW population *averaged linkage disequilibrium data* (physical distance, *r* ^2^ pairs) into *m*=100 equal size bins, s.t. each bin corresponds to a range of 2 kb (data only included LD for marker pairs at most 200 kb apart). The *r* ^2^ values in each bin were distributed as targets across the *m* columns in order, s.t. largest values were assigned to columns with the largest possible *r* ^2^ limits (given the columns’ allele frequencies).

Again, SimBA produced a good fit to the average LD distribution. The MAF distribution fits exactly, by design (see Corollary 1) and *F*_*st*_ fits very close to the target values.

### Comparison with existing methods

We compared SimBA with existing methods for generating populations with specific LD and MAF distributions: epiSIM [8], HapSim [6], and SIMLD [7]. epiSIM only considers the LD between adjacent markers, therefore its input consists of *r* ^2^ specifying the first distance only. HapSim is a perturbation approach, requiring as input an existing matrix having the desired distributions. SIMLD is a non-generative forward-simulator, starting from a fixed matrix having maximum possible LD and decaying it over generations. To test the accuracy of SimBA against the existing methods, we ran all methods simulating the JPT/CHB data, and we also checked the sensitivity by simulating the ASW and LWK populations. Let matrix *H* denote the first *m*=100 biallelic markers in chr 22 of the respective HapMap population, from which the MAF and *r* ^2^ constraints were extracted. Each method was run as follows.

epiSIM was given as input the minimum and maximum MAF, the mean *r* ^2^ for adjacent markers, and MAF and *r* ^2^ for the first two markers. HapSim was given as input the matrix *H*. SIMLD requires several population-specific parameters, which the authors have specified for the JPT/CHB population. Since the setting of their parameter values is not well defined and requires knowledge of population history, we used these same parameters for the ASW and LWK populations. SIMLD was given the MAF per marker and average *r* ^2^ per distance. SimBA was given as input the MAF per marker and *r* ^2^ per each pair of markers.

**Figure 5:**
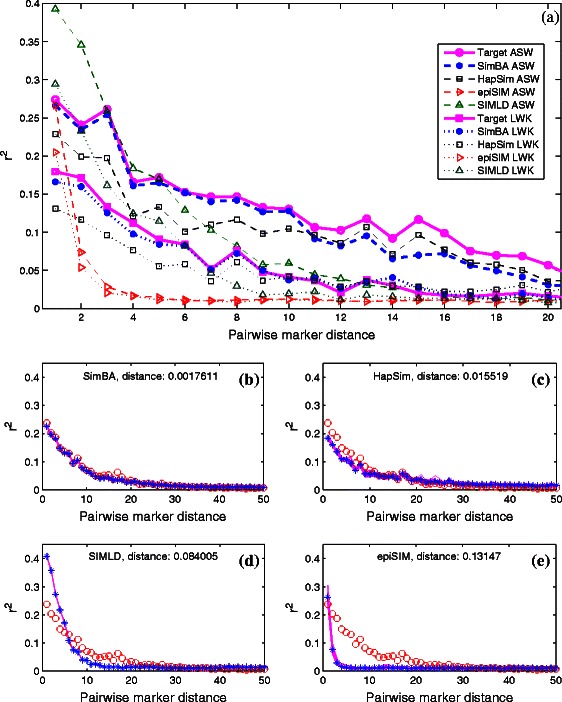
**SimBA comparison results.** **(a)** Sensitivity comparison with existing methods, comparing the results for two very similar populations ASW and LWK. SimBA hill climbing was run with *k*=6 for ASW, and *k*=10 for LWK. **(b-e)** Comparing existing methods on the JPT/CHB population. Results from 10 runs are shown as lines, while red “o” are the target and blue “*” are the average values across the runs. Sum of squared distances between average simulated and target values is shown for each method. SimBA hill climbing was run with *k*=10.

One of the characteristics of the output of SimBA is its high sensitivity as demonstrated in Figure 5(a). Note that two very similar *r* ^2^ distributions indeed resulted in distinct, correct distributions by SimBA, while the other methods fail to correctly match either of the two input distributions.

The JPT/CHB results across ten independent runs in Figure 5(b-e) show that only SimBA and HapSim constructed populations having LD close to the target values. SimBA is the most accurate, as measured by the sum of squared distances from target *r* ^2^ values. The run time of SimBA is better than most other methods, only SIMLD is faster. SIMLD was run with hard parameters specified for the JPT/CHB data by the authors, and these pre-tuned parameters produce a fairly accurate fit. epiSIM only matches the first distance since other distances are beyond the scope of the algorithm. HapSim results are only close to the target for the first two distances. The sensitivity comparison in Figure 5(a) shows that SimBA is far more accurate in distinguishing between the two similar populations than any other method. The other methods also produce two slightly different populations, but they are farther from the target values. HapSim does better on these populations than on the JPT/CHB data, while still being farther from the targets than SimBA.

To conclude the comparison study, epiSIM only considers the first distance, HapSim requires a sample matrix, and SIMLD requires optimizing several parameter values. All compared methods are less sensitive than SimBA in fitting the specified input distributions. The characteristics of SimBA compared to other methods are summarized in Table 1.

**Table 1:** **Comparison of population simulation methods**

	**epiSim**	**HapSim**	**SIMLD**	**SimBA**
Matches MAF distribution	-	X	X	X
Matches *r* ^2^ distribution	-	X	X	X
Number of control parameters	0	0	5	1
Population stratification	-	-	-	X
Simulation time	33	12	<1	5

SimBA matches the MAF and *r* ^2^ distributions for each marker, without requiring an exemplar population, and uses only one control parameter (*k*). Time (seconds) is the average time required for one run of the experiment shown in Figure 5(b-e). Experiments were conducted on the same x86_64 Linux Fedora system with 4-core 2.9 GHz processor and 16 GB RAM, except epiSim (requiring Matlab access) on a 64-bit Windows 7 system with 8-core 2.4 GHz processor and 8 GB RAM.

## Conclusions

SimBA is a novel non-generative method for simulating populations with various specified distributions such as MAF, LD etc. We show that SimBA outperforms the other methods both in terms of accuracy and efficiency. It runs in time linear with the size of the output. Furthermore, similar but distinct input characteristics result in distinct outputs with high fidelity to the specified distributions. SimBA does not require extensive population-specific parameter tuning or exemplar populations as starting points, unlike most non-generative methods. SimBA executable and manual are available at http://researcher.ibm.com/project/5669.
